# Subretinal delivery of adeno-associated virus serotype 2 results in minimal immune responses that allow repeat vector administration in immunocompetent mice

**DOI:** 10.1002/jgm.1327

**Published:** 2009-06

**Authors:** Susie E Barker, Cathryn A Broderick, Scott J Robbie, Yanai Duran, Mythili Natkunarajah, Prateek Buch, Kamaljit S Balaggan, Robert E MacLaren, James W B Bainbridge, Alexander J Smith, Robin R Ali

**Affiliations:** Division of Molecular Therapy, UCL Institute of OphthalmologyLondon, UK

**Keywords:** AAV, antibody, immune response, RPE65, subretinal

## Abstract

**Background:**

Adeno-associated virus serotype 2 (AAV2) vectors show considerable promise for ocular gene transfer. However, one potential barrier to efficacious long-term therapy is the development of immune responses against the vector or transgene product.

**Methods:**

We evaluated cellular and humoural responses in mice following both single and repeated subretinal administration of AAV2, and examined their effects on RPE65 and green fluorescent protein transgene expression.

**Results:**

Following subretinal administration of vector, splenocytes and T-cells from draining lymph nodes showed minimal activation following stimulation by co-culture with AAV2. Neutralizing antibodies (NAbs) were not detected in the ocular fluids of any mice receiving AAV2 or in the serum of mice receiving a lower dose. NAbs were present in the serum of a proportion of mice receiving a higher dose of the vector. Furthermore, no differences in immunoglobulin titre in serum or ocular fluids against RPE65 protein or AAV2 capsid between treated and control mice were detected. Histological examination showed no evidence of retinal toxicity or leukocyte infiltration compared to uninjected eyes. Repeat administration of low-dose AAV.hRPE65.hRPE65 to both eyes of *RPE65*^−/−^ mice resulted in transgene expression and functional rescue, but re-administration of high-dose AAV2 resulted in boosted NAb titres and variable transgene expression in the second injected eye.

**Conclusions:**

These data, which were obtained in mice, suggest that, following subretinal injection, immune responses to AAV2 are dose-dependent. Low-dose AAV2 is well tolerated in the eye, with minimal immune responses, and transgene expression after repeat administration of vector is achievable. Higher doses lead to the expression of NAbs that reduce the efficacy of repeated vector administration. Copyright © 2009 John Wiley & Sons, Ltd.

## Introduction

Adeno-associated viruses (AAV) are parvoviruses that have not been associated with any pathogenicity in humans 1. Twelve serotypes have now been identified 2–4 and these show tropism for many different cell types 2. Studies using AAV serotype 2 (AAV2) have demonstrated that the vector has considerable promise for gene transfer to the liver, muscle and eye, and it has been used to treat animal models of several inherited conditions, such as muscular dystrophy 5, haemophilia 6 and several types of inherited retinal degeneration 7. These pre-clinical studies have demonstrated that effective treatment of these disorders might be possible 8–10. On the whole, however, these trials have not demonstrated the level of efficacy observed in pre-clinical animal studies, and it appears that this might be due to host immune responses to either the gene transfer vector or the transgene product 11. Because the vast majority of the population are exposed to wild-type AAV in the first decade of life, patients may have pre-existing immunity to AAV. It is estimated that up to 80% of the population have circulating antibodies against AAV2 12, whereas 30–50% have antibodies with neutralizing activity 13,14. This pre-existing immunity may inhibit transgene expression, and any boost to an anti-vector immune response may render future re-administration of the vector ineffective. Another potential barrier to efficacious long-term therapy is the development of immune responses against the transgene product.

Several detailed studies on the immune responses occurring following AAV-mediated gene delivery in animal models have been carried out, but have generated some conflicting reports and remain inconclusive, with immune responses appearing to depend on the route of administration, vector dose and species differences 15. Immune responses against AAV vectors leading to a reduction of transgene expression have been observed in dogs following intramuscular administration 16. Here, a T-cell response against AAV2 and AAV6 vector capsid proteins was observed, regardless of the transgene expressed, the muscle type injected or the cellular specificity of the promoter. By contrast, another canine muscular dystrophy model showed that T-cell infiltration was dependent on the transgene product; intramuscular administration of AAV2 vectors expressing β-galactosidase resulted in intense T-cell infiltration, whereas AAV2 expressing no transgene resulted in no cellular infiltration 17, despite similar doses being used in each study. Studies in mice, however, have demonstrated that hepatic gene transfer can achieve transgene-specific tolerance through the induction of regulatory T cells, even at high doses of vector 18, whereas lower doses can result in long-term transgene expression following intramuscular administration with no destruction of transduced cells 19.

The high level of pre-exposure to wild-type AAV in the human population has led to problems in clinical trials of AAV gene transfer 11. However, when AAV has been administered to an immune privileged site such as the central nervous system (CNS), a much more limited response against the vector has been observed systemically, with only a minority of patients developing neutralizing antibodies (NAbs) and no anti-vector antibodies being detected in the CNS itself 10. The eye is also well known to be an immune privileged site and therefore intraocular administration of AAV may result in a different pattern of immune responses compared to other routes. The mechanism of this immune privilege is multifaceted and is the result of several mechanisms, including physical barriers such as the blood–retinal barrier, immunological ignorance and peripheral tolerance of eye-derived antigens 20. An intraocular immunosuppressive microenvironment utilizes mechanisms such as FasL expression on the corneal endothelium 21 and the retinal pigment epithelium (RPE) 22 and immune deviation in both the anterior chamber 23 and the vitreous cavity 24. Immune privilege might afford the eye a level of protection, to a certain degree, against damaging inflammatory responses, therefore protecting and preserving the cells crucial to vision that are unable to repair damage or regenerate in adult differentiated tissue. Long-term AAV-mediated gene expression in the mouse eye has previously been demonstrated in several studies 25,26, suggesting that the vector is well tolerated.

There have been extensive investigations into immune responses against AAV following administration to the liver or muscle. By contrast to the prevalence of NAbs in humans, the frequency of existing memory T-cells is very low in the normal healthy population 14. However, T-cell responses against AAV2 have been observed transiently in a clinical trial following the intrahepatic delivery of AAV2, where an expanded capsid-specific CD8 + effector population was identified. This led to the destruction of transduced hepatocytes in one patient and a substantial increase in neutralizing antibody levels was observed in all patients 9. However, to date, there does not appear to have been any detailed analysis of cellular and humoural immune responses following intraocular administration of AAV, and what effect these responses may have on repeat administration of vector; this information is critical because ocular gene therapy clinical trials are now underway to treat Leber's congenital amaurosis, a form of severe early onset retinal degeneration caused by mutations in *RPE65* 27,28. *RPE65* encodes a 65-kDa protein that is a vital component in the visual cycle. Clinical trials have demonstrated that a single subretinal injection of AAV2 encoding human *RPE65* can mediate a significant improvement in visual function, even in patients with advanced loss of vision. One study used a hybrid constitutive cytomegalovirus (CMV)/chicken β-actin promoter to drive the expression of hRPE65 and administered a dose of 1.5 × 10^10^ vector genomes 28. In this trial, patients were transiently immunosuppressed with corticosteroids and no cellular responses against AAV2 or RPE65, or anti-RPE65 humoural responses were detected, although one patient did show an increase in NAbs against AAV2. The other trial administered a dose of 1 × 10^11^ vector genomes of an AAV2 vector containing the endogenous hRPE65 promoter to drive hRPE65 cDNA expression 27, thereby restricting expression of the transgene to RPE cells only. Patients were also transiently immunosuppressed with corticosteroids in this trial. No cellular responses against the AAV2 capsid were detected, and no changes in humoural responses against RPE65 or AAV2 compared to pre-gene therapy levels were detected in any patients. Therefore, in both studies, the vector appears to be well tolerated in patients with missense mutations who are transiently immunosuppressed, with evidence of minimal inflammation or immune responses against the vector or transgene product. However, it has not yet been determined what effect previous subretinal exposure to the AAV2 vector has on the level of transgene expression following repeated subretinal injection or the effect that vector re-administration has on immune responses against the transgene product or the vector itself, particularly in the absence of immunosuppression. In the present study, we undertook a thorough evaluation of the cellular and humoural response in non-immunosuppressed mice following administration of AAV2 encoding an endogenous retinal protein (hRPE65) or a reporter gene encoding an exogenous reporter (green fluorescent protein, GFP), and we examined in detail the effects of these immune responses on transgene expression or the repeated administration of vector.

## Materials and methods

### Animals

Female 4–6 weeks old C57BL/6 mice (Harlan, UK) or *rd12*^−/−^ (*RPE65*^−/−^) mice 29 were used for all experiments. All animals were maintained humanely with institutional approval and in accordance with the ARVO Statement for the Use of Animals in Ophthalmic and Vision Research. At the end of the experiments, mice were euthanased by an overdose of pentobarbital.

### Viral vectors

AAV2 vectors contained either the human RPE65 cDNA under the control of the human RPE65 promoter (AAV.hRPE65.hRPE65), or the GFP reporter gene under the control of the CMV intermediate-early promoter (AAV.CMV.GFP). All vectors contained the AAV-2 inverted terminal repeats. The AAV.hRPE65.hRPE65 vector was supplied by Targeted Genetics (Seattle, WA, USA). The AAV.CMV.GFP was produced by transient transfection of BHK cells with the pD10-CMV-GFP vector together with pHAV7.3 that contains the *Rep* and *Cap* genes. HSV-DISC was used as the helper virus as described previously 30. Seventy-two hours post-transfection, cells were harvested and pelleted. The pellets were freeze-thawed with vortexing and the crude lysate was filtered sequentially through 5 µm and 0.8 µm filters. The cleared lysate was applied to a heparin-agarose column (Sigma, Poole, UK) equilibrated with phosphate-buffered saline (PBS) + 2.5 mM KCl + 1 mM MgCl_2_ (PBS-MK). The bound virus was washed with 10 ml of PBS-MK + 0.1 M NaCl and eluted with 6 ml of PBS-MK + 0.4 M NaCl. The eluate was concentrated by spinning through Centricon-10 (Millipore, Watford, UK) columns down to a final volume of approximately 200–250 µl. Virus was titred by dot-blot to determine the number of vector genomes per millilitre by comparison to a standard curve generated by serial dilutions of plasmid. Vector concentration was then adjusted to 1 × 10^11^ or 5 × 10^11^ vg/ml.

### Subretinal injection of viral vectors

For all experiments, mice were anaesthetized with an intraperitoneal injection of 0.15 ml of a mixture of Dormitor (medetomidine hydrochloride, 1 mg/ml; Pfizer Pharmaceuticals, Sandwich, UK) and ketamine (100 mg/ml; Fort Dodge Animal Health, Southampton, UK) mixed with sterile water for injections in the ratio 5 : 3:42. The pupils of all animals were dilated using topical 1% tropicamide and 2.5% phenylepherine (Chauvin Pharmaceuticals, Romford, UK). Viral vector injections into the subretinal space were performed through the peripheral cornea under the direct control of a surgical microscope with the tip of a 10-mm 34-gauge hypodermic needle mounted on a 5 µl syringe (Hamilton AG, Bonaduz, Switzerland). 2 × 2 µl of vector suspension was then injected subretinally containing 1 × 10^11^ or 5 × 10^11^ vg/ml. Anaesthesia was reversed using 0.15 ml of Antisedan (atipamezole hydrochloride, 0.10 mg/ml; Pfizer) and all animals received chloramphenicol 1% eye ointment to the cornea.

### Histological assessment of disease

Enucleated eyes were fixed in 4% paraformaldehyde, embedded in optimal cutting temperature (OCT) compound (Tissue-Tek, Sakura Finetek, The Netherlands) and cryosectioned into 8–10-µm vertical serial sections through the papillary-optic nerve axis and then stained with haematoxylin and eosin. Sections were coded and the number of inflammatory cells in the anterior and posterior segments was counted. Four histological sections per eye were evaluated. The inflammatory cells were counted under ×40 magnification in five fields, each of the anterior and posterior chambers (each field corresponds to 0.2 mm^2^, verified with the grid of a Neubauer haemocytometer).

### Assessment of local immune cell priming to AAV2

Mice received subretinal injections of 2 × 2 µl of 1 × 10^11^ or 5 × 10^11^ vg/ml. Seven days post-injection, mice were euthanased and single cell suspensions were prepared from the draining cervical lymph nodes. Cells were cultured in triplicate for 48 h in 96-well round bottomed plates (2.5 × 10^5^ cells/well) in the presence of AAV.hRPE65.hRPE65 at two dilutions (2 × 10^4^ and 5 × 10^3^ vg/ml), 2.5 µg/ml Concanavilin A (ConA) (Sigma) or media alone. Cells were then pulsed with 0.5 µCi of [^3^H]-thymidine and harvested onto glass fibre mats 16 h later. Cell proliferation was measured using a β-scintillation counter (Perkin Elmer, Waltham, MA, USA). For analysis by flow cytometry, single cell suspensions from draining cervical lymph nodes were incubated with antibody for 1 h a 4 °C [CD25-FITC, CD4-PE (Pharmingen, Oxford, UK), CD69-FITC (Invitrogen, Paisley, UK)]. Cells were then washed, fixed in 4% paraformaldehyde (PFA) in PBS and analysed on an EPICS XL flow cytometer (Beckman Coulter, High Wycombe, UK).

### Assessment of systemic immune responses to AAV22

Mice were received subretinal injections of 2 × 2 µl of 1 × 10^11^ or 5 × 10^11^ vg/ml. Fourteen days post-injection, mice were euthanased and single cell suspensions of splenocytes were prepared. Cells were cultured in triplicate for 48 h in 96-well round bottomed plates (2.5 × 10^5^ cells/well) in the presence of AAV.hRPE65.hRPE65 at three dilutions (2 × 10^4^, 1 × 10^4^ and 5 × 10^3^ vg/ml), 2.5 µg/ml ConA (Sigma) or media alone. T-cell proliferation assays were performed as described above. Supernatants from triplicate wells of cultured cells were harvested and cytokine expression [interferon (IFN)γ and interleukin (IL)-4] by lymphocytes in response to stimulus was determined by Duoset enzyme-linked immunosorbent assay (ELISA) (R&D Systems, Abingdon, UK) according to the manufacturer's instructions.

### Detection of NAbs to AAV2

Mice received subretinal injections of 2 × 2 µl of 1 × 10^11^ or 5 × 10^11^ vg/ml. Three weeks post-injection, mice were euthanased and blood was harvested by cardiac puncture and allowed to clot on ice. Samples were then spun down and serum stored at −80 °C. Ocular fluids were obtained by removal of fluid from freshly enucleated eyes by piercing the anterior chamber with a needle and drawing fluid out with a capillary tube. Fluids were stored at −80 °C until use. To determine NAb titres, serial dilutions of serum were prepared in triplicate and 1 × 10^8^ vg of AAV.CMV.GFP was added to each sample. Plates were incubated at 37 °C for 1 h, then the contents of the wells were added to 96-well plates of 293T cells containing 2.5 × 10^4^ cells per well. Plates were incubated for 48 h and then the number of GFP^+^ cells per well was counted using an inverted fluorescence microscope. For the ocular fluid samples, the amount of starting material was limited and therefore all samples were diluted 1 : 30 in PBS before assay. The titre of NAb was defined as the highest dilution that produced 50% fluorescence compared to the media only control.

### Detection of total immunoglobulin (Ig)A, IgG and IgM to AAV2 and recombinant RPE65

Ocular fluid and serum samples were prepared as above at 3 and 6 weeks post-subretinal injection. 96-well Maxisorp microtitre plates (NUNC, Roskilde, Denmark) were coated with AAV.hRPE65.hRPE65 (50 000 vg/well in 100 µl of PBS) or recombinant human RPE65 (gift from Professor Martin Warren, University of Kent, Canterbury, UK) (1 : 5000 dilution in 100 µl of PBS) overnight at room temperature, then washed with PBS + 0.05% Tween-20 (PBS-T). Plates were blocked with 1% bovine serum albumin in PBS for ≥1 h at room temperature, then washed and samples (1 : 50 or 1 : 100 dilution) applied. Plates were incubated at room temperature for 1.5 h and then washed three times with PBS-T. Bound mouse Ig was detected with anti-mouse IgA,G,M-HRP (AdB Serotec, Kidlington, UK) for 1.5 h at room temperature, then washed three times with PBS-T and colour was developed with TMB substrate (Pharmingen, Oxford, UK) and quenched with 1 N HCL. Absorbance at 450 nm was quantified using a plate reader (E Max, Molecular Devices, Wokingham, UK).

### Electroretinography

Six-week-old *rd12*^−/−^ mice (*RPE65*^−/−^) received 2 × 2 µl AAV.hRPE65.hRPE65 (1 × 10^11^ vg/ml) in a subretinal injection in the right eye. Three weeks later the left eye also received 2 × 2 µl AAV.hRPE65.hRPE65 (1 × 10^11^ vg/ml) via subretinal injection. Six and 9 weeks after the first injection, scotopic electroretinograms (ERGs) were performed. ERGs from injected animals were recorded in a standardized fashion, as previously described 25. Animals were dark-adapted overnight and Ganzfeld ERGs were obtained simultaneously from both eyes to provide an internal control. Mice were anaesthetized and dilated as described above. A single drop of 2% hydroxypropylmethylcellulose was placed on each cornea to keep it moistened. ERGs were recorded using commercially available equipment (Toennies Multiliner Vision; Jaeger/Toennies, Germany) after corneal contact electrodes and midline subdermal reference and ground electrodes were placed. Single flash recordings were obtained at light intensities increasing from 0.1–3000 mcds/m^2^. Ten responses per intensity level were averaged with an interstimulus interval of 5 s (0.1, 1, 10 and 100 mcds/m^2^) or five responses per intensity with a 17-s interval (1000 and 3000 mcds/m^2^).

### *In vivo* GFP expression

Mice received a subretinal injection in the right eye of 2 × 2 µl of 5 × 10^11^ vg/ml AAV.CMV.GFP. Three weeks later, they received a subretinal injection in the left eye of 2 × 2 µl of 5 × 10^11^ vg/ml AAV.CMV.GFP. Five weeks after the second injection, the mice were culled and eyes enucleated and fixed in 4% PFA. Eyes were embedded in OCT and 8–10-µm cryosections were cut. Sections were counterstained with propidium iodide and imaged using Q-capture Pro software (Q-Imaging, St Helens, UK).

### Statistical analysis

A comparison between group ELISAs and T-cell proliferations was performed using two-tailed *t*-tests. A comparison between b-wave amplitudes for ERG data was performed using two-tailed paired *t*-test. In both cases, *p* < 0.05 was considered statistically significant.

## Results

### No early local inflammatory response following subretinal administration of AAV2

**Figure 1:**
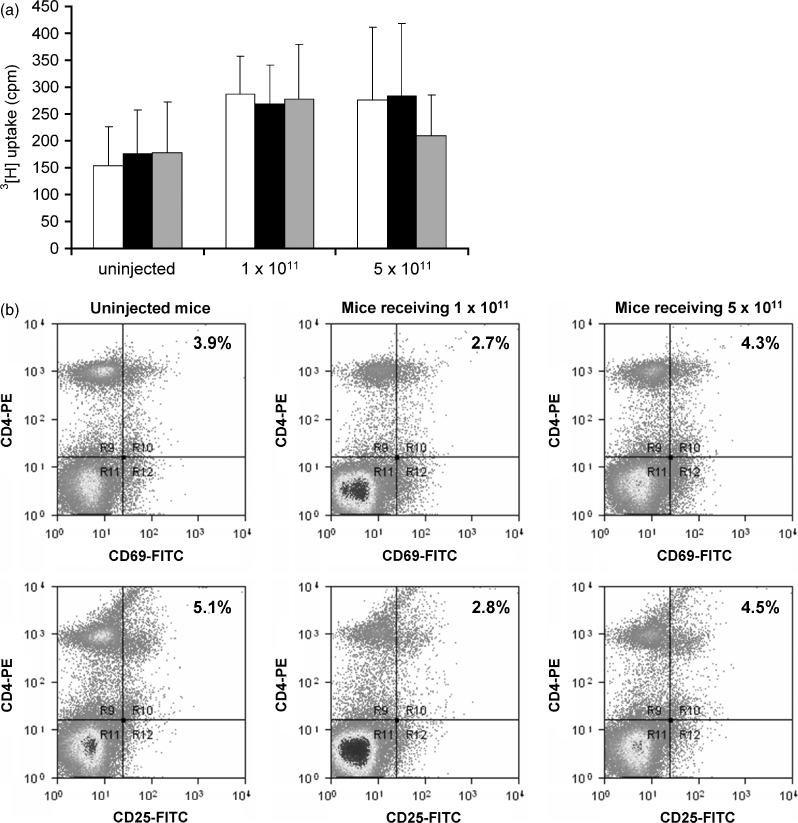
Local immune responses of cells from draining lymph nodes 7 days after subretinal administration of AAV.hRPE65.hRPE65. (a) Cells from low-dose (2 × 2 µl of 1 × 10^11^ vg/ml) and high-dose (2 × 2 µl of 5 × 10^11^ vg/ml) AAV-injected mice or na*ï*ve uninjected controls were assayed for proliferation following incubation with 5000 or 20 000 vg/ml AAV.hRPE65.hRPE65. Negative control wells contained cells incubated in media alone. All wells were incubated for 48 h and then pulsed with [^3^H]-thymidine for 16 h, then harvested. All test wells co-cultured with AAV.hRPE65.hRPE65 showed no significant increase in proliferation compared to media-only controls (*p* > 0.05), and comparable responses to AAV.hRPE65.hRPE65 were observed between the injected and uninjected control groups (*p* > 0.05). White bars, media-only control; black bars, 5000 vg/ml; grey bars, 20 000 vg/ml. Positive control wells were stimulated with ConA and showed cpm of >25 000, indicating that the cells were functional (data not shown). Results are expressed as the mean ± SEM cpm. (b) Lymph node cells from low-dose (2 × 2 µl of 1 × 10^11^ vg/ml) and high-dose (2 × 2 µl of 5 × 10^11^ vg/ml) AAV-injected mice or na*ï*ve controls were incubated with AAV.hRPE65.hRPE65 for 48 h and then stained for the presence of the surface bound T-cell activation markers CD25 or CD69 together with CD4. No upregulation of activation markers was detectable on cells from injected mice compared to na*ï*ve controls

To determine whether a local cellular immune response was initiated to AAV2 capsid proteins following subretinal injection, C57Bl/6 mice (*n* = 6 per group) received a subretinal injection of either 2 × 2 µl of 1 × 10^11^ or 5 × 10^11^ vg/ml of an AAV2 vector carrying the human RPE65 cDNA driven by a human RPE65 promoter 27 (AAV.hRPE65.hRPE65). A control group consisted of uninjected C57Bl/6 mice. All animals were na*ï*ve to AAV before the experiment. Lymphocyte suspensions were prepared from the cervical lymph nodes 7 days after subretinal administration. Because transgene expression is undetectable at 1 week post-injection, any immune responses detected should be against the vector itself. Cells from the draining lymph nodes were then analysed to detect AAV-specific T-cells. Lymphocytes were rechallenged *in vitro* with AAV.hRPE65.hRPE65 and [^3^H]-thymidine-incorporation assays indicated that there was no significant proliferation of T-cells from injected mice in response to co-culture with any of the dilutions of AAV.hRPE65.hRPE65 compared to the negative media-only control (*p* > 0.05) (Figure 1a). Also, there was no significant difference in proliferation between cells from treated and uninjected control mice (*p* > 0.05). Lymphocytes treated with ConA served as a positive control for the T-cell proliferation assay, in which all samples demonstrated counts per minute of >25 000 (data not shown).

Flow cytometry analysis was performed to detect any upregulation of CD25 or CD69 on CD4^+^ T-cells from the draining lymph nodes of injected versus control mice. The IL-2 receptor-α chain, CD25, is known to be expressed on regulatory T-cells. We have studied CD25 expression here as an activation marker because this molecule is not expressed on na*ï*ve unstimulated T-cells, but is upregulated on na*ï*ve lymphocytes following stimulation 31–33, whereas the early membrane receptor CD69 is expressed following lymphocyte activation but is not detected on resting lymphocytes 34,35. An increase in these markers would suggest that the cells were responding to antigen. Cells from injected mice showed no difference in the expression levels of CD25 or CD69 on CD4^+^ T-cells compared to cells from control mice, indicating that the cells are not activated following co-culture with AAV2 (Figure 1b).

Eyes were taken from all mice and haematoxylin and eosin stained histological sections were examined for any cellular infiltrate around the injection site. There was no gross evidence of infiltrating cells (data not shown).

### No early systemic immune responses following subretinal administration of AAV2

**Figure 2:**
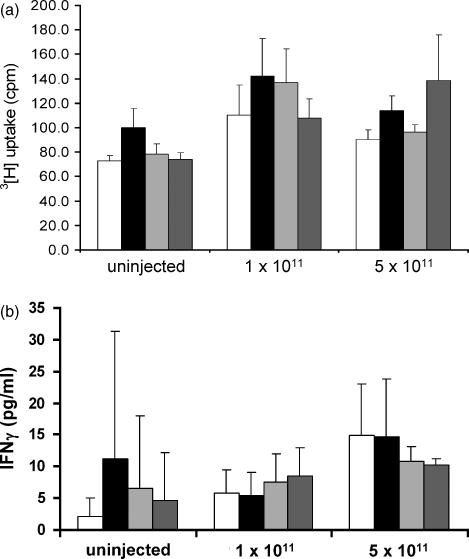
Systemic immune responses from splenocytes 14 days after subretinal vector administration of AAV.hRPE65.hRPE65. (a) Splenocytes from mice injected with low-dose (2 × 2 µl of 1 × 10^11^ vg/ml) and high-dose (2 × 2 µl of 5 × 10^11^ vg/ml) vector and na*ï*ve controls were tested for proliferation following co-culture with dilutions (5000, 10 000 or 20 000 vg/ml) of AAV.hRPE65.hRPE65. Negative control wells contained cells incubated in media alone. Cells were incubated for 48 h and then pulsed with [^3^H]-thymidine for 16 h and harvested. All test wells incubated with AAV.hRPE65.hRPE65 showed no significant increase in proliferation compared to unstimulated controls (*p* > 0.05), and no significant difference was observed between the proliferation of injected and uninjected control groups (*p* > 0.05). White bars, media-only control; black bars, 5000 vg/ml; light grey bars, 10 000 vg/ml; dark grey bars, 20 000 vg/ml. Positive control wells were stimulated with ConA and showed a cpm of >25 000, indicating that cells were functional (data not shown). Results are expressed as the mean ± SEM cpm. (b) IFNγ expression of splenocytes following co-incubation with AAV.hRPE65.hRPE65. Splenocytes from mice injected subretinally with low-dose (2 × 2 µl of 1 × 10^11^ vg/ml) or high-dose (2 × 2 µl of 5 × 10^11^ vg/ml) vector or na*ï*ve uninjected controls were assayed for IFNγ expression following co-culture with dilutions (5000, 10 000 or 20 000 vg/ml) of AAV.hRPE65.hRPE65. Negative control wells contained cells incubated in media alone. Cells were incubated for 48 h and then supernatants were harvested and assayed by ELISA. There was no significant difference in IFNγ expression by T-cells taken from either injected mice or uninjected controls (*p* > 0.05), indicating that the splenocytes were not responding to AAV2. White bars, media-only control; black bars, 5000 vg/ml; light grey bars, 10 000 vg/ml; dark grey bars, 20 000 vg/ml. The supernatant from ConA-treated splenocytes was used as a positive control in the ELISA in which all samples demonstrated IFNγ secretion of >3750 pg/ml (data not shown). Results are expressed as the mean ± SEM

To investigate whether subretinal delivery induced a systemic immune response against AAV2 capsid, C57Bl/6 mice (*n* = 6 per group) received either 2 × 2 µl of 1 × 10^11^ or 5 × 10^11^ vg/ml AAV.hRPE65.hRPE65 into the subretinal space. A control group consisted of uninjected C57Bl/6 mice. All animals were na*ï*ve to AAV before the experiment. Because transgene expression is negligible at 2 weeks post-injection, we were looking for immune responses only against the vector itself. Fourteen days after subretinal injection, single cell suspensions of splenocytes were analysed to detect AAV2-specific T-cells. Splenocytes were rechallenged *in vitro* with AAV2 and proliferation assays were performed. No significant increase in proliferation of T-cells from injected mice with either dose of AAV.hRPE65.hRPE65 were detected in response to co-culture with AAV2 compared to the negative media-only control (*p* > 0.05) (Figure 2a). Also, there was no significant difference in proliferation between cells from treated and uninjected control mice (*p* > 0.05). ConA-treated splenocytes were used as a positive control, in which all samples demonstrated counts per minute of >25 000 (data not shown), showing that the cells were functional.

Supernatants from the splenocytes cultured with AAV.hRPE65.hRPE65 were also analysed for production of T_h_1 (IFNγ) or T_h_2 (IL-4) cytokines. No significant differences in the concentration of either cytokine were detected in the culture medium following incubation with AAV2 compared to media-only negative controls (*p* > 0.05). Also, there was no significant difference in IFNγ (*p* > 0.05) (Figure 2b) or IL-4 (data not shown) expression in T-cells taken from either injected mice or uninjected controls, indicating that the splenocytes were not responding to AAV2. The supernatant from ConA-treated splenocytes was used as a positive control in which all samples demonstrated IFNγ secretion of >3750 pg/ml (data not shown), showing that the cells were functional.

Histological sections from the eyes from all mice were also analysed for any cellular infiltrate. There was no gross evidence of infiltrating cells (data not shown).

### No humoural responses (IgA, IgG or IgM) against human RPE65 or AAV2 following subretinal delivery of AAV.hRPE65.hRPE65

**Figure 3:**
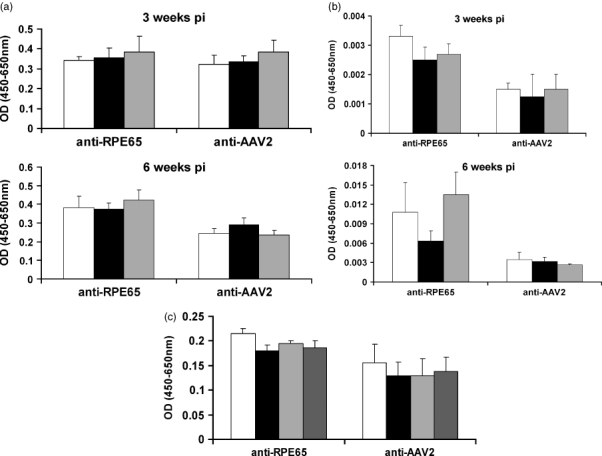
Total IgA, IgG and IgM in C57Bl/6 (wt) and *rd12*^−/−^ (*RPE65*^−/−^) mice that received subretinal administration of AAV.hRPE65.hRPE65 (2 × 2 µl of 1 × 10^11^ or 5 × 10^11^ vg/ml) and uninjected controls. Serum (a) and ocular fluids (b) from C57Bl/6 mice were harvested 3 or 6 weeks following vector administration and were assayed by ELISA for antibodies against AAV2 capsid and hRPE65 protein. No significant difference in the total immunoglobulin level to AAV2 capsid or hRPE65 in serum or ocular fluids was observed between the mice that received vector and the uninjected control group at either time point (*p* > 0.05). White bars, uninjected control mice; black bars, mice injected with 2 × 2 µl of 1 × 10^11^ vg/ml; grey bars, mice injected with 2 × 2 µl of 5 × 10^11^ vg/ml. (c) AAV.hRPE65.hRPE65 was injected subretinally into *rd12*^−/−^ mice (2 × 2 µl of 1 × 10^11^ vg/ml in right eye on day 0 and in left eye on day 21). Serum was harvested at days 0, 21, 42 and 63 and assayed by ELISA for antibodies against AAV2 capsid or hRPE65. No significant change in antibody level to AAV2 vector (*p* > 0.05) or hRPE65 protein (*p* > 0.05) was detected following the first or repeated subretinal administration. White bars, day 0; black bars, day 21; light grey bars, day 42; dark grey bars, day 63. Results are expressed as the mean ± SEM OD_450_

To investigate whether subretinal delivery induced a humoural immune response against AAV2 capsid or hRPE65, C57Bl/6 mice (*n* = 6 per group) received a subretinal injection of either 2 × 2 µl of 1 × 10^11^ or 5 × 10^11^ vg/ml AAV.hRPE65.hRPE65. A group of uninjected mice served as controls. The total serum IgA, IgG and IgM against AAV2 or hRPE65 at 3 weeks post-injection was then determined by ELISA. Equivalent cohorts of mice were also examined at 6 weeks post-injection. No significant difference in circulating antibody levels was detected between the groups of mice receiving the two different doses of vector (*p* > 0.05), and treated mice showed no significant difference compared to uninjected control animals for either AAV2 or hRPE65 antibodies at either time point (*p* > 0.05) (Figure 3a), suggesting that there was no increase in circulating antibodies against either vector or transgene.

Ocular fluid samples were also harvested to detect local levels of antibody at the site of transgene expression and were analysed by ELISA for IgA, IgG and IgM against AAV2 or hRPE65 at 3 and 6 weeks post-injection. Again, no significant difference in antibody levels against AAV2 or hRPE65 was detectable compared to controls at either time point (*p* > 0.05) (Figure 3b). This experiment was performed in mice that express endogenous RPE65. Although our vector encodes the human form of the protein, RPE65 is very highly conserved across species, with 95% homology between the human and murine forms 36, reducing the likelihood of being recognized as nonself in wild-type mice. We also tested whether a humoural response to hRPE65 would occur following administration of vector to *rd12*^−/−^ animals, which lack endogenous expression of the RPE65 protein. *Rd12*^−/−^ mice (*n* = 6) received subretinal injections of 2 × 2 µl of 1 × 10^11^ vg/ml AAV.hRPE65.hRPE65 in the right eye and 21 days later in the left eye. Serum samples were harvested on days 0, 21, 42 and 63 and total IgA, IgG and IgM was assayed by ELISA. No significant increase in serum Ig specific for hRPE65 was detected compared to the pre-injection baseline (*p* > 0.05) (Figure 3c).

### Limited presence of NAbs to AAV2 following single subretinal administration in C57Bl/6 mice

**Figure 4:**
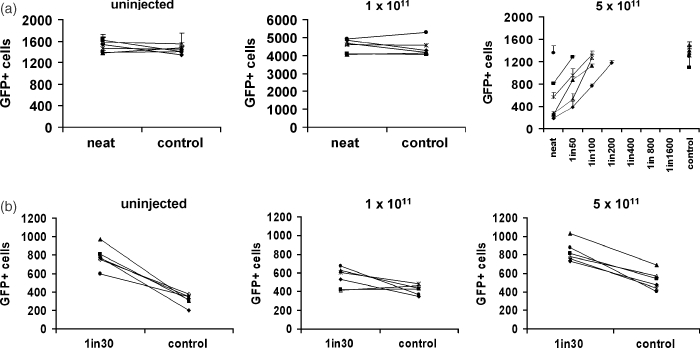
Neutralizing antibody titres in mice 3 weeks after subretinal administration of low-dose (2 × 2 µl of 1 × 10^11^ vg/ml) or high-dose (2 × 2 µl of 5 × 10^11^ vg/ml) AAV.hRPE65.hRPE65. The neutralizing antibody titre in (a) serum and (b) ocular fluids was determined by the ability to inhibit the infection of 293T cells with AAV.CMV.GFP. Each line represents a separate mouse. There was no significant decrease (*p* > 0.05) in GFP^+^ cells following incubation with serum from mice in the uninjected group or mice receiving the lower dose of AAV.hRPE65.hRPE65 compared to no sera control wells, and thus no NAbs were detected. In the group of mice receiving the higher dose of virus, there was a significant decrease in the number of green cells in the neat serum sample compared to the no sera control (*p* = 0.003) and also a significant decrease compared to neat serum from uninjected mice (*p* = 0.0007). NAbs were present five out of six mice receiving the high dose of AAV.hRPE65.hRPE65; for three of these five mice, the NAb titre (50% inhibition) was 1 : 50 dilution and, for two of the mice, the NAb titre was 1 : 75. No NAbs were detected in the ocular fluids of any injected or na*ï*ve uninjected control mice

One of the major barriers to successful AAV-mediated gene therapy is the presence of NAbs, which bind to the vector particles, inhibiting vector uptake into the cell and which also prevent effective re-administration of vector. We therefore tested for the presence of NAbs against AAV2 capsid proteins in the serum and ocular fluids from injected C57Bl/6 mice. AAV.CMV.GFP was pre-incubated with neat and serial dilutions of serum harvested 3 weeks after subretinal injection of AAV.hRPE65.hRPE65 from the mice described in the group above, or at a 1 : 30 dilution of the ocular fluids (as the volume of fluid obtained was a limiting factor) and then applied to 293T cells. After 48 h, the number of GFP^+^ cells was quantified and compared with control wells that received AAV.CMV.GFP but no serum or ocular fluids. We did not detect any significant decrease (*p* > 0.05) in GFP^+^ cells compared to control wells, and thus no increase in NAb titre in the mice from the uninjected group or the group receiving the 2 × 2 µl of 1 × 10^11^ vg/ml dose (Figure 4a). In the group of mice receiving the 2 × 2 µl of 5 × 10^11^ vg/ml dose of virus, there was a significant decrease in the number of green cells in the neat serum sample compared to the media control (*p* = 0.003) and also a significant decrease compared to neat serum from uninjected mice (*p* = 0.0007). In the high-dose group, five out of six mice showed the presence of NAbs (Figure 4a). For three of these five mice, the NAb titre (50% inhibition) was 1 : 50 dilution and, for two of the mice, the NAb titre was 1 : 75. This suggests that the development of NAbs against AAV2 may be dependent on the dose of injected vector.

No NAbs were detected in wells incubated with the ocular fluids from any of the injected mice at either dose compared to the ocular fluids from uninjected controls or media-only wells (*p* > 0.05) (Figure 4b). Importantly, even the mice that had detectable circulating NAbs against AAV2 in their serum showed no evidence of NAbs within the eye.

Histological sections from eyes of all mice were also analysed for any cellular infiltrate. There was no gross evidence of infiltrating cells was detected (data not shown).

### Repeated subretinal administration of low-dose AAV *in vivo* results in functional rescue

**Figure 5:**
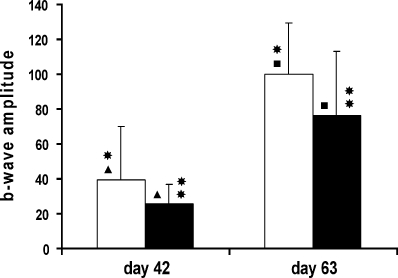
Electroretinography of mice injected with low-dose AAV2. Six-week-old *rd12*^−/−^ (*RPE65*^−/−^) mice were injected with 2 × 2 µl of 1 × 10^11^ vg/ml AAV.hRPE65.hRPE65 in the right eye and then, 3 weeks later, 2 × 2 µl of 1 × 10^11^ vg/ml AAV.hRPE65.hRPE65 was administered into the left eye. Six weeks after the initial injection, scotopic ERG showed that b-wave amplitudes increased in both eyes compared to uninjected controls (data not shown). Nine weeks after subretinal injection, b-wave amplitudes continued to increase significantly (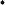: *p* = 0.006, right eye, 6 versus 9 weeks; 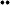: *p* = 0.04, left eye, 6 versus 9 weeks), demonstrating rescue in both eyes, which indicated that vector from the second injection was not neutralized and that hRPE65 was expressed in both eyes. There was no significant difference in b-wave amplitude between the right and left eyes at either day 42 post-injection (▴ : *p* = 0.27) or day 63 post-injection (▪ : *p* = 0.25)

For the treatment of some ocular disorders, it might be necessary to deliver an AAV2 vector on more than one occasion, particularly when it is necessary to treat both eyes. In mice, AAV-mediated gene replacement therapy for RPE65 defects has been previously shown to restore function following subretinal administration 37. To determine whether administration of vector in the second eye might be as effective as the first injection, 6-week-old *rd12*^−/−^ mice (*n* = 6) received subretinal administration of 2 × 2 µl of 1 × 10^11^ vg/ml AAV.hRPE65.hRPE65 in the right eye and, 3 weeks later, an equivalent injection in the left eye. Scotopic ERGs were performed 6 and 9 weeks after the initial injection. The mean b-wave amplitudes of both treated eyes were increased 6 weeks following vector delivery compared to untreated *rd12*^−/−^ mice (data not shown), and also continued to increase significantly by 9 weeks post-injection (*p* = 0.006, right eye, 6 weeks versus 9 weeks; *p* = 0.04, left eye, 6 weeks versus 9 weeks), indicating that the vector from the second injection was not neutralized, resulting in hRPE65 expression in both eyes (Figure 5). There was no significant difference in b-wave amplitude between the right and left eyes at either day 42 post-injection (*p* = 0.27) or day 63 post-injection (*p* = 0.25).

### Repeated delivery of high-dose AAV *in vivo* results in high transgene expression at the first injection site but variable expression at the second site of vector administration

**Figure 6:**
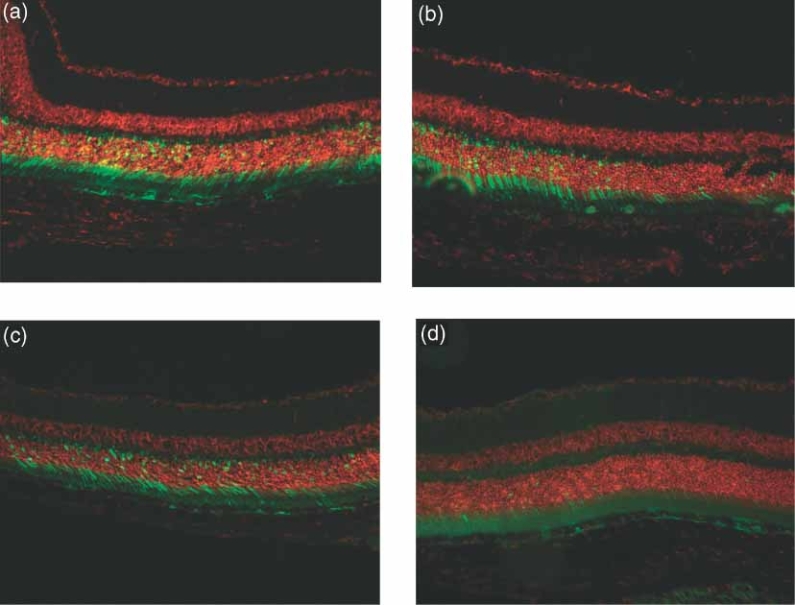
Fluorescence microscopy of GFP expression following re-administration of high-dose AAV2. AAV.CMV.GFP (2 × 2 µl of 5 × 10^11^ vg/ml) was administered subretinally in to the right eye of C57Bl/6 mice (*n* = 8). Three weeks later, 2 × 2 µl of 5 × 10^11^ vg/ml AAV.CMV.GFP was injected subretinally into the left eye of the same group of mice. Five weeks after the second injection, mice were culled and eyes cryosectioned and counterstained with propidium iodide. (a, b) GFP expression was detected in all right eyes 8 weeks after the first subretinal injection. Five weeks after the second subretinal injection into the left eye, GFP expression in the eye that received the second injection was variable. Three out of eight mice had as many GFP^+^ cells in the second injected eye as the eye that received the first injection (c), and the remaining mice had much lower levels of transduced cells in the second injected eye (d), demonstrating that repeat administration of vector and transgene expression is achievable in a proportion of eyes. Representative images are shown

The presence of NAbs has been shown to prevent repeated administration of AAV2 vectors via certain routes of delivery. To determine whether successful repeat administration of high-dose AAV2 was possible, a dose that can induce the production of NAbs, 2 × 2 µl of 5 × 10^11^ vg/ml AAV.CMV.GFP, was administered into the subretinal space of the right eye of wild-type C57Bl/6 mice (*n* = 8). Three weeks later, 2 × 2 µl of 5 × 10^11^ vg/ml AAV.CMV.GFP was administered into the subretinal space of the left eye. Five weeks after the second administration, the eyes were enucleated and sectioned. There was no evidence of gross infiltration of leukocytes in any eyes. High levels of transgene expression were observed in all the eyes that had received the first injection (Figures 6a and 6b). However, expression of GFP was variable in the eyes that received the second injection. Three out of eight mice had as many GFP^+^ cells in the second injected eye as the eye that received the first injection (Figure 6c), whereas five out of eight had much lower numbers of GFP^+^ cells in the second injected eye (Figure 6d). Analysis of serum from the mice in this cohort that received repeated administration of high-dose vector showed high NAb titres of between 1 : 2000–1 : 5000 (data not shown) compared to NAb titres of up to 1 : 75 in the mice receiving the single injection of high-dose vector (Figure 4a). There did not, however, appear to be a correlation between the NAb titre and the level of GFP expression in the second injected eye (i.e. the animals with the lowest NAb titres did not have the highest GFP expression). These data suggest that the subretinal delivery of high doses of AAV2 induces the production of NAbs, with the second injection boosting titres that may inhibit transgene expression from subsequent administrations of AAV2 at a distant site.

## Discussion

In the present study, we investigated the immune response to AAV2 vectors following subretinal delivery in immunocompetent mice. The main aim of the study was to evaluate the humoural and cellular responses to the vector or transgene after vector administration and to determine whether this impacted on repeated vector delivery. To our knowledge, this is the first study to investigate both cellular and humoural responses following AAV2 delivered to the subretinal space. We show functional rescue of the ERG in the *rd12*^−/−^ mouse with AAV2.hRPE65.hRPE65 was observed following injection of vector into both eyes 3 weeks apart. This demonstrates that the principle that vector of the same serotype and expressing the same transgene can be re-administered effectively.

Over recent years, immune responses against gene delivery vectors have become one of the most important issues in the field of gene therapy. It has been established previously that, although adenoviral vectors induce strong immune responses that severely inhibit the efficacy of repeated vector administration, AAV vectors are much less immunogenic 38. However, more recent studies have shown that AAV is capable of stimulating an immune response that can be detrimental to gene delivery 9,16, although the route of administration and vector dose appear to be key elements in determining the degree of anti-AAV immunity that is generated.

Our data show that subretinal administration of a lower dose (2 × 2 µl of 1 × 10^11^ vg/ml) AAV2 elicits minimal immune responses. NAbs were only detected in the serum of mice receiving the higher dose (2 × 2 µl of 5 × 10^11^ vg/ml) of AAV but not at the lower dose, suggesting that the development of NAbs are dependent on the dose of vector, which is in agreement with other studies 19,39. Furthermore, no NAbs were detected in the ocular fluids of mice receiving the higher dose. Importantly, no cellular infiltrate was observed in any eyes following AAV injection, even in those mice with circulating NAbs. This is in agreement with previous studies investigating ocular gene delivery 40, but contrasts with other routes of administration such as intramuscular 16 and portal vein injection 41. Other studies, however, show that inflammation is not due the AAV vector, but rather is dependent on the transgene 17,42.

By contrast to the low-dose re-administration, when a higher dose of vector was injected into the right and then the left eye 3 weeks apart, boosted NAb titres inhibited the transgene expression in a proportion of the eyes that received the second injection. The transgene expression from the eye that had received the first injection remained high in all animals. This suggests that the dose of vector delivered is a critical factor in the development of anti-vector immune responses, which has a substantial impact of the efficacy of subsequent administration of vector. The source of the variation in transgene expression remains to be established. This has important implications for the development of clinical gene delivery protocols.

In the future, it may be advantageous to engineer vectors that lack immunogenic motifs. The heparin sulphate proteoglycan (HSPG) motif on the VP3 capsid protein of AAV2 has been shown to be responsible for uptake into dendritic cells, leading to the activation of capsid specific CTLs. When this sequence was mutated to ablate HSPG binding, the immune response was attenuated, yet the tropism of the vector was unchanged. Furthermore, AAV serotypes that do not express HSPG binding motifs appeared inherently less immunogenic 43. Other studies have shown that a non-HSPG-binding mutant of AAV2 showed detargeting from the spleen and liver compared to wild-type AAV2, leading to higher levels of the non-HSPG-binding AAV2 mutant remaining circulating in the blood following intravenous injection 44.

Several studies have shown that NAbs against AAV2 do not exhibit cross reactivity against other serotypes, allowing the possibility of re-administration with other AAV serotypes; this ‘cross-administration’ approach has shown efficacy following intramuscular gene delivery 15,19. Several different AAV serotypes are able to transduce the retina, so it may be possible to utilize this approach for effective re-administration in the eye. Greater immune responses have been observed in large animal models than in mice that have received the equivalent vector dose. Dogs appear to be particularly susceptible, but transient immunosuppression has been used to reduce immune responses, permitting long-term transgene expression 45,46.

In conclusion, our data show that subretinal delivery of AAV2 in mice is well tolerated, even in the absence of immunosuppression. No cellular immune response was observed to the vector in any mice. Mice receiving the lower dose did not develop NAbs, whereas a proportion of the mice receiving the single high-dose of vector developed NAbs, and those receiving repeated administration of high-dose vector all developed the highest titres of NAbs, suggesting that the induction of NAbs is dependent on: (i) the initial dose of vector and (ii) NAb titres are boosted by repeat exposure to vector. Crucially, in the present study, we showed that lower doses vector could be re-administered and that repeat injections of vector were successful and demonstrated therapeutic efficacy. This is important because, if both eyes of a patient are to receive gene therapy, they are unlikely to be injected at the same time. Therefore, the demonstration that the same type of vector can be administered subretinally at a later time-point is vital to the development of a protocol requiring a second administration of vector. Although the data obtained in the present study suggest that immunosuppression may not be necessary for lower vector doses, it may allow increased doses of vector to be delivered and thus reduce the risk of immune responses still further.
